# Embryo-specific expression of a visual reporter gene as a selection system for citrus transformation

**DOI:** 10.1371/journal.pone.0190413

**Published:** 2018-01-02

**Authors:** Manjul Dutt, Flavia T. Zambon, Lígia Erpen, Leonardo Soriano, Jude Grosser

**Affiliations:** 1 Citrus Research and Education Center, University of Florida, Lake Alfred, Florida, United States of America; 2 Universidade de São Paulo, Escola Superior de Agricultura Luiz de Queiroz, Piracicaba, São Paulo, Brazil; NARO Institute of Fruit Tree Science, JAPAN

## Abstract

The embryo-specific *Dc3* gene promoter driving the *VvMybA1* anthocyanin regulatory gene was used to develop a visual selection system for the genetic transformation of citrus. *Agrobacterium*-mediated transformation of cell suspension cultures resulted in the production of purple transgenic somatic embryos that could be easily separated from the green non-transgenic embryos. The somatic embryos produced phenotypically normal plants devoid of any visual purple coloration. These results were also confirmed using protoplast transformation. There was minimal gene expression in unstressed one-year-old transgenic lines. Cold and drought stress did not have any effect on gene expression, while exogenous ABA and NaCl application resulted in a minor change in gene expression in several transgenic lines. When gas exchange was measured in intact leaves, the transgenic lines were similar to controls under the same environment. Our results provide conclusive evidence for the utilization of a plant-derived, embryo-specific visual reporter system for the genetic transformation of citrus. Such a system could aid in the development of an all-plant, consumer-friendly GM citrus tree.

## Introduction

In plants, the 5’ flanking regions of most protein-coding genes contain DNA sequences that interact with the basic transcription initiation complexes and various transcription factors [1]. These DNA sequences regulating the function of the downstream gene are termed promoters. Most plant promoters are composed of three essential components: the transcription start site, the TATA box, and several hundred sequences that include upstream activating sequences (UASs), enhancers, upstream repressing sequences (URSs), and silencers [2]. Promoters are important elements used in genetic engineering, and they can either direct gene expression uniformly in most tissues and cells [3–5] or target expression to a specific tissue [6–8]. Targeted gene expression in a transgenic plant by utilizing a tissue specific promoter can result in enhanced transgene protein production in the organ of interest and does not cause a metabolic drain in the overall plant [9, 10]. This is because many tissue-specific promoters can provide tightly regulated gene expression in only the tissue of interest [11–13].

Seed-specific promoters target the gene expression in the seed tissues. Several seed-specific promoters and their cis-elements have been characterized [14–16], and a number of them have been utilized to target the transgene product to different parts of the seed. Metabolic engineering using these promoters have allowed scientists to increase the plant’s nutritional content by upregulating oil production and the manipulation of phenolic compounds for better grain quality and yield, resulting in a number of improved transgenic cultivars [17, 18]. Studies on the molecular and cellular events during the critical stages of early embryogenesis and seed development have resulted in the generation of a wealth of knowledge on the function of some seed-specific promoters [8, 19, 20].

Citrus transformation studies have mainly utilized constitutive promoters to express both the transgene of interest and the selectable marker gene (usually an antibiotic resistance marker) with or without a reporter gene in the genome of the plant [21–23]. Reporter genes code for proteins that can be easily detectable and are therefore particularly useful for selection systems. Green fluorescent protein (GFP), β-glucuronidase (GUS) and luciferase (LUC) are the most commonly used reporter genes in plant transformation [24]. Some studies have also exploited anthocyanin accumulation as a visual marker in cereal and fruit transformation [25–27]. Anthocyanins are endogenous pigments that are responsible for the red, purple and blue colors in flowering plants. The anthocyanin reporter system is nondestructive, does not require an exogeneous substrate, or produce a toxic compound and has no related environmental or health concerns [24].

Unlike the gene of interest, selectable marker/reporter gene(s) are important mainly in the early stages of genetic transformation. Consequently, the constitutive expression of these genes is unnecessary in the regenerated plants and can even be associated with substantial fitness penalties [28]. In addition, development of transgenic plants that have the selectable marker/reporter gene(s) either excised from the system or switched off can alleviate public concerns on the environmental safety of genetically modified plants [29]. Marker-free plants have been produced in citrus, mainly through site specific recombination [30–32] or PCR analyses of all regenerated plantlets [33]. These procedures are either very time consuming or result in a small population of successfully excised transgenic lines.

Accordingly, it is beneficial to develop a robust selection system for marker-free transformation of citrus. Using tissue-specific or inducible promoters in association with reporter genes may overcome the shortcomings of constitutive expression. In this report, we outline a novel method of selectable reporter gene silencing by utilizing the tightly regulated embryo-specific promoter of the carrot *Dc3* gene, which is highly expressed during the initial phases of embryogenesis [34], coupled with a visual anthocyanin reporter gene (the *Vitis vinifera*-derived *VvMybA1* [35]), following transformation of citrus using either cell suspension cultures or protoplasts to regenerate reporter gene expression-free citrus plants.

## Materials and methods

### Construction of plant transformation vectors

The 1.5 kb *Dc3* promoter [34] in pBluescript KS and cloned between the *Pst*I and *Hin*dIII sites was a kind gift from Dr. Terry Thomas (Dept. of Biology, Texas A&M University, College Station, TX). The promoter fragment was amplified utilizing primers DC3-F and DC3-R (Table 1) and the Phusion® Hot Start Flex 2X Master Mix (New England Biolabs, Ipswich, MA) to introduce a *Hin*dIII restriction site at the 5’ end and a *Bam*HI site at the 3’ end. Two constructs were produced for this study: the first utilizing a modified pCAMBIA1300 binary plasmid for *Agrobacterium*-mediated transformation and the second utilizing a modified pUC18 *Escherichia coli* cloning vector.

**Table 1:** Primer sequences used in this study.

Target gene	Primer	Purpose	Primer sequence 5’→ 3’
*Dc3*	DC3-F	Cloning	AAGCTTTGCTGTACCATATCTTTGTAGCC
	DC3-R		GGATCCGGTGGCTTTCTTTGCAGATGT
*VvmybA1*	VVM-F	qPCR	CCAGGAAGAAGGGAGAGATAAAC
	VVM-R		CTAACAGGCTTTCCCACCATA
*GAPC*	GAPC-F	qPCR	GGAAGGTCAAGATCGGAATCAA
	GAPC-R		CGTCCCTCTGCAAGATGACTCT

The Sanger-sequence-verified *Dc3* amplicon was cloned by replacing the 35S promoter at complementary sites of the pCAM1300-VvMyb to produce the plasmid pCAMDC3-VvMyb (S1 Fig). A pUC18-based intermediate cloning vector previously constructed to contain the *VvMybA1* transgene (GenBank accession no. AB097923 [35]) under the control of a 35S promoter and a terminator sequence of the *Pisum sativum-*derived RuBisCO small subunit (rbcS) was modified to replace the 35S promoter with the *DC3* promoter (pUDC3-VvMyb; S1 Fig).

### Initiation of cell suspension cultures

Embryogenic callus from *Citrus sinensis* (L.) Osbeck cv. ‘Hamlin’ was initiated from unfertilized ovules as outlined earlier [22]. Cell suspension cultures were initiated from actively dividing one-year-old embryogenic callus (S2 Fig). Five grams of embryogenic callus was incubated in 25 ml of liquid H + H cell proliferation medium and sub-cultured on a 2-week transfer cycle [36]. Cells were harvested for transformation between 5 to 7 days after subculture.

### *Agrobacterium*-mediated transformation of cell suspension cultures

Five milliliters of a vigorously growing *Agrobacterium* culture (EHA105; [37]) initiated the night before containing the pCAMDC3-VvMyb construct was seeded into 45 ml YEP medium containing appropriate antibiotics and allowed to grow for an additional 3 hours [38]. One gram of suspension cells were harvested and subsequently incubated in a 0.3-OD *Agrobacterium* suspension that had been re-suspended in DOG medium (EME sucrose supplemented with 5 mg L^-1^ kinetin) [39] for 20 minutes. Cells were subsequently blotted dry on sterile Whatman filter paper disks (GE Healthcare, Chicago, IL), plated on solid DOG medium supplemented with 100 μM acetosyringone, and incubated in the dark at 25°C for 5 days before transfer to EME medium supplemented with maltose (EME-M) for selection. The EME-M medium was supplemented with 400 mg L^-1^ timentin (Duchefa Biochemie B.V., Netherlands) and 25 mg L^-1^ hygromycin B. After a month of culture, the cells were overlaid with 2 ml of a 1:2 (v:v) mixture of 0.6 M BH3 and 0.15 M EME-M liquid media [39]. Anthocyanin-overexpressing globular-stage somatic embryos were individually cultured over 0.22-mm cellulose acetate membrane filters on embryo maturation EME-M medium [40] before transfer into B+ germination medium [36]. Well-developed PCR positive plantlets were transferred for further root development by transferring into RMAN rooting medium [36]. Plantlets that did not root were micrografted [38]. Well-acclimated plants after a year of transfer to the greenhouse were utilized for all subsequent molecular analyses.

### Protoplast-mediated transformation

Plasmid DNA containing the pUDC3-VvMyb construct was utilized for all protoplast transformation experiments. Cell suspension cultures were used for the isolation of protoplasts essentially as described earlier [41]. Enzymatically digested protoplasts obtained from 1 gram of cells were purified by centrifugation on a sucrose-mannitol gradient (S2 Fig) and suspended in 0.6 M BH3 protoplast culture medium to a final concentration of 2 × 10^6^ cells ml^-1^ [36]. Then, 20 μg of plasmid DNA suspended in sterile water (2 mg ml^-1^ concentration) was added to a 15-ml polystyrene culture test tube (Thermo Fisher Scientific Inc, Waltham, MA) containing 0.25 ml of the protoplast suspension. The mixture was incubated for 10 minutes followed by the addition of 0.5 ml of a 40% polyethylene glycol (PEG) solution. After a 30-minute incubation, four drops of the PEG-protoplast mixture were pipetted into the center of a 60 mm × 15 mm Petri dish. PEG elution and protoplast washing were carried out as described earlier [36]. The washed protoplasts were cultured in a liquid medium containing a 1:1 mixture of 0.6 M BH3 protoplast culture medium with 0.6 M EME medium [36] at 25–27°C under diffused light for 4–6 weeks. Transgenic cells were visualized with the naked eye. Purple colored transgenic somatic embryos were separated from the non-transgenic green embryos and placed in embryo maturation and germination medium, essentially in a manner similar to that described for the cell suspension transformation process. *In vitro* plantlets were tested by PCR, and the positive plants acclimated in a similar manner as those obtained from the *Agrobacterium*-mediated transformation and were utilized for subsequent molecular analyses.

### Molecular analysis of transformants

Initial verification of transgenic status was performed on *in vitro* leaves. Leaves were harvested from putative transgenic plants and non-transgenic control and verified by PCR utilizing the Extract-N-Amp™ Plant PCR Kit (Sigma-Aldrich, St. Louis, MO) and gene-specific primers [42]. Total RNA was isolated utilizing the RNeasy Mini Kit (Qiagen Inc. Valencia, CA) according to the manufacturer’s protocol. A 1-μg aliquot of RNA was reverse transcribed using RevertAid First-Strand cDNA Synthesis Kit (Thermo Fisher Scientific Inc). Real-time quantitative PCR (qPCR) was performed using the StepOne Plus system (Thermo Fisher Scientific Inc) utilizing SYBR Green reagents. The relative mRNA levels were compared to those of the *Citrus sinensis* glyceraldehyde-3-phosphate dehydrogenase (GAPC) gene [43] and calculated using the 2^-ΔΔCT^ method [44]. Citrus genomic DNA from one-year-old transgenic trees and non-transgenic control was isolated using the PureLink™ Genomic Plant DNA Purification Kit (Thermo Fisher Scientific Inc). Transgene presence was re-confirmed by PCR. The transgene copy number in the selected lines was also validated using qPCR, essentially as previously described [45, 46]. All primer sequences used in this study are listed in Table 1.

### Stress analyses

Two node cuttings from seven randomly selected year old transgenic lines (five from the *Agrobacterium*-mediated transformation and two from the protoplast transformation) were rooted in a mist bed [47]. Six-month-old well-rooted cuttings containing a minimum of 4–6 leaves and growing in 5 × 18 cm Deepot cells (Stuewe and Sons, Inc. Tangent, Oregon) were used for subsequent experiments. All stress treatments were conducted in a Percival Scientific (Percival Scientific Inc., Perry, Iowa) growth chamber under controlled conditions. The light intensity in the growth chamber as measured 15 cm from the lamps was 300 μmol m^-2^ s^-1^. For cold stress experiments, plants were kept at 8° C ± 2° C for 7 days before evaluation. Plants were drought stressed by withholding irrigation and maintained under these conditions until leaves had fully wilted (5 days). Exogenous ABA ((±)-cis,trans-abscisic acid, Sigma, St. Louis, MO) was applied by spraying the cuttings with 10 ml of 100 μM (±)ABA (Fluka) in 0.5% (v/v) Tween 20. Two drenches of 200 mM NaCl were applied at a weekly interval before leaf sampling.

### Leaf gas exchange measurements

Seven selected transgenic lines along with a non-transgenic control were subjected to leaf gas exchange measurements. All plants were watered to soil field capacity one day prior to measurement. Measurements were taken on the same day in the morning (11 am-12:30 pm) with the quantum of external light averaging 329.35 μmol m^-2^ s^-1^. The leaf temperatures at the time of measurement were 40.46 ± 1.38°C, and air temperatures were 38.72 ± 0.63°C. Three distinct fully expanded leaves were utilized for the physiological responses of net assimilation of CO_2_ (A, μmol m^-2^ s^-1^), transpiration rate (E, mol m^-2^ s^-1^), stomatal conductance to water vapor (gsw, mol m^-2^ s^-1^) and intercellular CO_2_ (Ci, μmol mol^-1^). Measurements were conducted with a portable photosynthesis system (LI-6800; LICOR Inc., Lincoln, NE). Instantaneous water use efficiency (WUE_instantaneous_; μmol CO_2,_ mmol H_2_O^-1^) was calculated by the ratio of net assimilation and transpiration rate [48].

### Data analysis and statistics

The data were analyzed by one-way analysis of variance (ANOVA, JMP^®^ Pro, Version 12.2.0., SAS Institute Inc., Cary, NC) as a completely randomized design. Significant differences in mean values were separated by pair comparisons using Tukey-Kramer HSD with α = 0.05. Bar graphs represent the mean of each line for each parameter, and error bars represent the standard deviation from the mean.

## Results

### The *Dc3* promoter is suitable for embryo-specific gene expression in citrus

**Fig 1:**
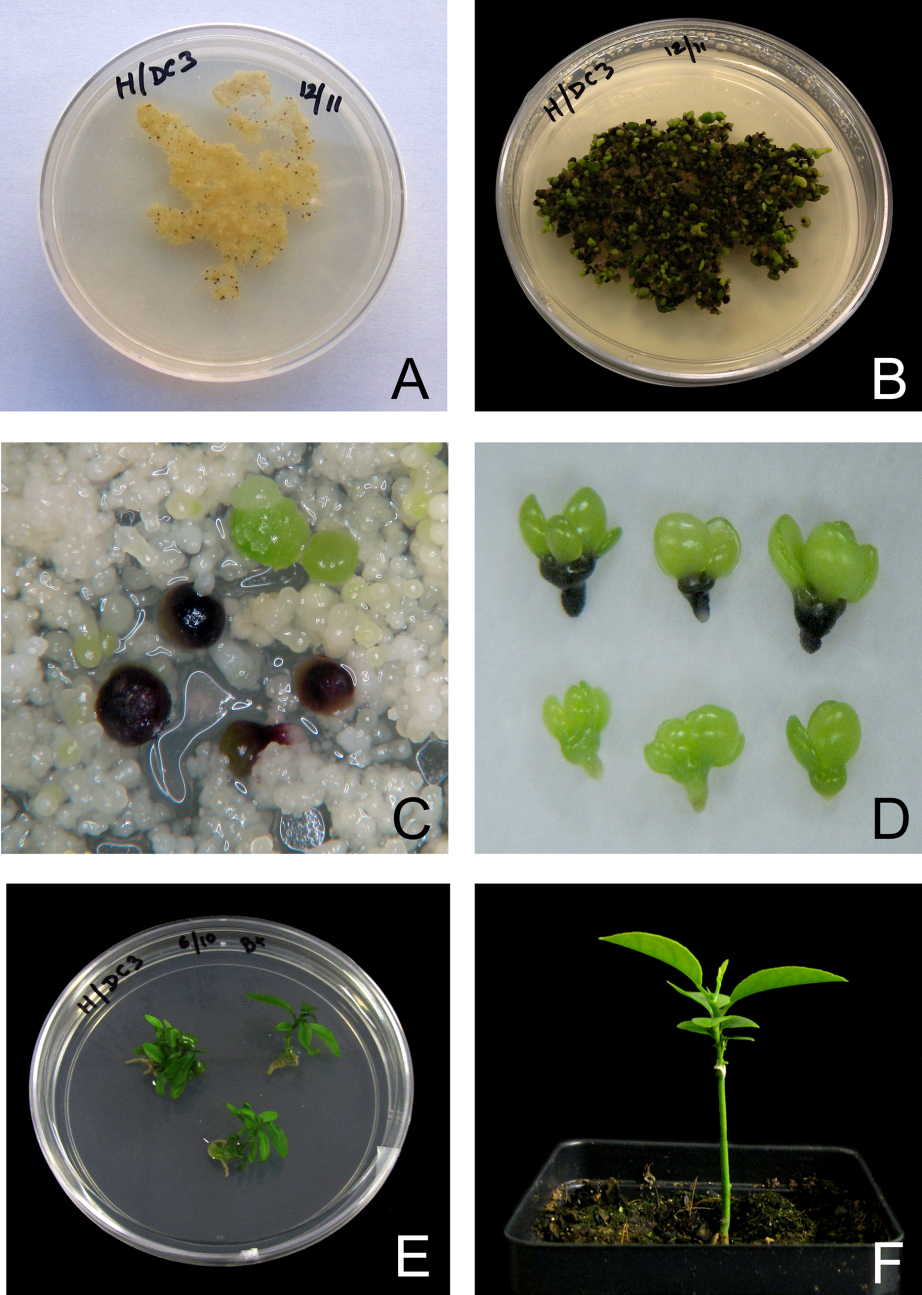
Steps in the *Agrobacterium* mediated transformation of citrus suspension cells with a construct containing the *VvmybA1* gene driven by the *Dc3* embryo-specific promoter. A) Citrus cell suspension cultures on EME-M medium at the beginning of embryonic development; B) Anthocyanin producing transgenic somatic embryos after 60 days in EME-M medium; C) Anthocyanin expression in transgenic (purple) embryos with non-transgenic (green) embryos; D) Anthocyanin free cotyledon development in transgenic embryos (top) and non-transgenic embryos at similar stage of development (bottom); E) Transgenic embryos germinating in the plant regeneration medium (B+ medium); F) A normal regenerated transgenic plant without any visual purple coloration (no *VvmybA1* gene expression) micro grafted on Carrizo rootstock.

The present study was conducted to develop a simple selectable marker system for the genetic transformation of citrus, utilizing an embryo-specific, plant-derived visual reporter gene system. Initial studies utilizing an *Agrobacterium* mediated transformation protocol was conducted to test the suitability of the *Dc3* promoter in citrus. *Agrobacterium*-mediated transformation of cell suspension cultures utilizing the pCAMDC3-VvMyb construct produced 18 ‘Hamlin’ sweet orange transgenic lines. Each line was confirmed for the presence of the transgene twice—first *in vitro* and subsequently after acclimatization in the greenhouse. Two lines tested negative in the second PCR confirmation step (S3 Fig). The cells did not exhibit any visual coloration following transformation. After two months in the selection medium, purple embryos were produced (Fig 1A and 1B). Globular stage transgenic embryos could be easily identified from the non-transgenic escapes based on their coloration (Fig 1C) and 56 individual embryos were manually selected and individually placed in maturation medium (Table 2). All germinating embryos gradually began to lose coloration and 18 phenotypically normal transgenic lines were regenerated. None of the developing cotyledons had the characteristic purple coloration derived from expression of the *VvMybA1* transgene (Fig 1D). All lines that regenerated into normal plants were phenotypically normal and devoid of any visual purple coloration (Fig 1E and 1F). Transgenic lines remained visually free of reporter gene expression in the greenhouse.

**Table 2:** Transformation efficiency of ‘Hamlin’ sweet orange from 1 gram of suspension cell derived cultures.

Transformation system	VvmybA1-negative embryos ^a^	VvmybA1-positive embryos ^a^	VvmybA1-positive shoots (Plant recovery efficiency (%))^b^
Suspension Cells	74	56	18 (32)
Protoplast	194	21	11 (52)

^a^ Data from three independent experiments. Results indicate the total number of embryos produced (VvmybA1 positive and negative)

^b^ Plant recovery efficiency was calculated as the total number of VvmybA1-positive shoots/total number of VvmybA1-positive embryos produced X 100.

### The *Dc3* promoter can be utilized to produce transgenic plants using protoplast transformation

**Fig 2:**
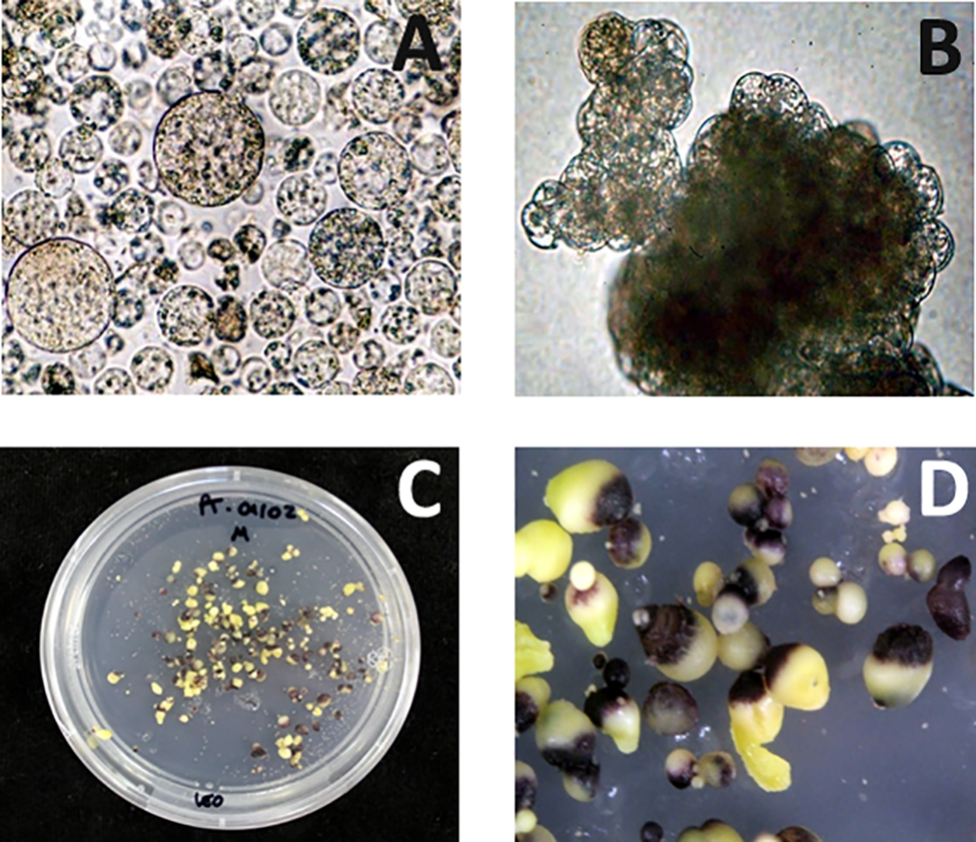
Steps in the protoplast transformation process with a construct containing the *VvmybA1* gene driven by the *Dc3* embryo-specific promoter. A) Transformed protoplast derived cells producing anthocyanins; B) Micro-calli derived from protoplasts; C) Anthocyanin producing somatic embryos; D) Closeup of the regenerating anthocyanin producing somatic embryos.

In contrast to *Agrobacterium*-mediated transformation where transient gene expression could not be detected, transient *VvMybA1-*expressing cells following protoplast transformation utilizing the plasmid DNA containing the pUDC3-VvMyb construct could be identified within 3 days following transformation (Fig 2). Primary selection was based on the purple coloration produced as a result of *VvMybA1* gene expression. Eleven phenotypically normal plants were regenerated using this system from a total of 21 purple embryos that were placed on maturation medium (Table 2). Similar to that observed in the cell suspension transformation system, we were able to produce transgenic plants that had the marker gene switched off in the developing plantlets.

### Expression level of *VvMybA1* in one-year-old transgenic lines

**Fig 3:**
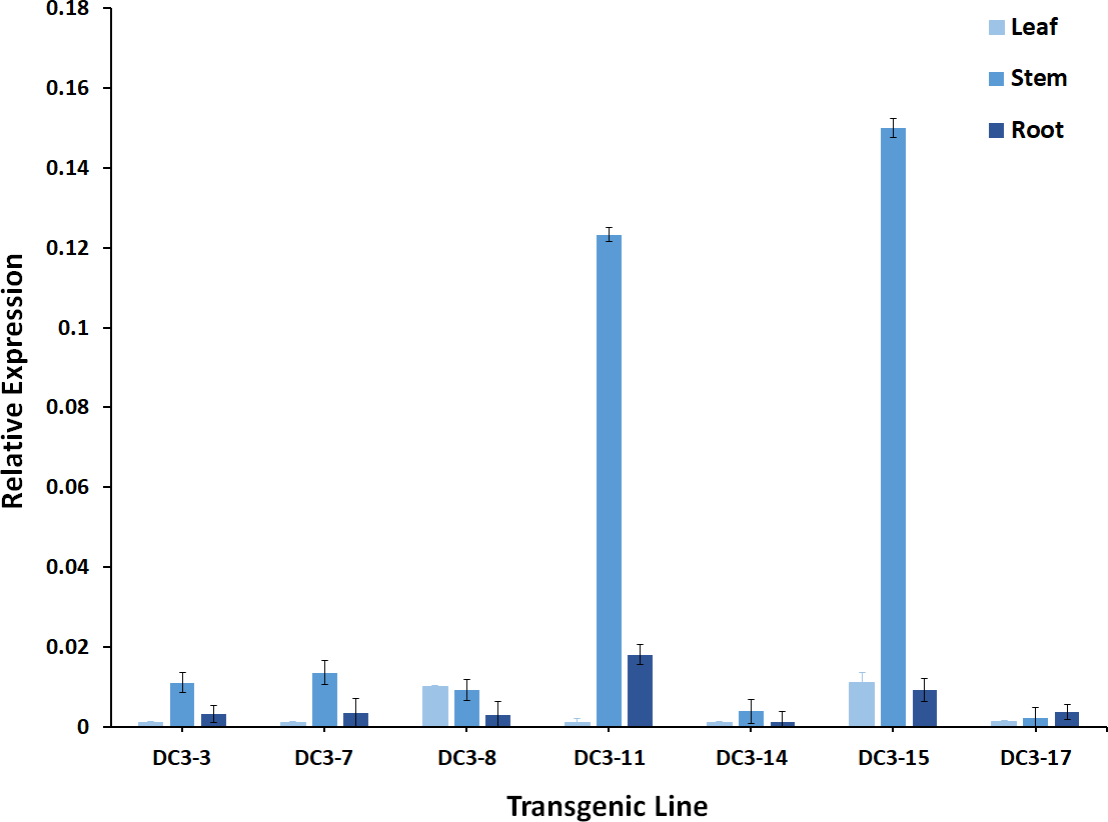
Relative *VvMybA1* gene expression in leaves, stem and roots in selected transgenic lines.

PCR confirmed the transgenic ‘Hamlin’ sweet orange lines regenerated following either the *Agrobacterium*-mediated transformation protocol or the protoplast protocol had the *VvMybA1* transgene stably incorporated into the genome (S3 Fig). Transgene copy number validated using qPCR ranged from 1 to 4 copies in all the PCR positive lines (data not presented), and there was no significant variation in plants regenerated from either method. The copy numbers of the 7 selected lines evaluated in this study (5 lines obtained through the *Agrobacterium* mediated transformation method and 2 through the protoplast transformation method) are presented in Table 3 and ranged from 1–3. *VvMybA1* transgene levels were analyzed in each of these lines. Transgene expression could not be detected in the leaves of most transgenic lines apart from DC3-8 and DC3-15 (0.01-fold). Transgene expression could however be detected in some transgenic stems. However, the results were variable and depended on the transgenic line evaluated. The relative gene expression was minimal and ranged from 0.02-fold (DC3-17) to 0.15-fold (DC3-15). *VvMybA1* expression was negligible in the roots and could not be detected in most lines (Fig 3). DC3-11 had a 0.18-fold change in the expression level when compared to the non-transgenic control.

**Table 3:** Transgene copy number determination using quantitative real-time PCR by comparison of transgenic lines with external plasmid controls.

Transgenic Line	Obtained using	Mean Cp ^A^	STD Cp ^B^	Copy number ^C^
DC3-3	Agrobacterium	28.051	0.196	2
DC3-7	Agrobacterium	27.834	0.368	2
DC3-8	Agrobacterium	29.263	0.157	1
DC3-11	Agrobacterium	28.930	0.0489	1
DC3-14	Agrobacterium	28.441	0.0810	2
DC3-15	Protoplast	29.047	0.263	1
DC3-17	Protoplast	27.514	0.104	3
Plasmid-1C^D^	-	29.433	0.128	1
Plasmid-2C	-	28.506	0.141	2
Plasmid-3C	-	27.704	0.111	3
Plasmid-4C	-	27.276	0.213	4
Plasmid-5C	-	26.927	0.157	5

^A^ Average values of crossing point from three sample replicates.

^B^ Standard deviations.

^C^ Approximate copy number derived from the average values of extrapolated concentration relative to a single transgene copy.

^D^ Plasmid DNA used for copy number calculations

### *Dc3* promoter in the transgenic plants respond variably to different abiotic stresses

**Fig 4:**
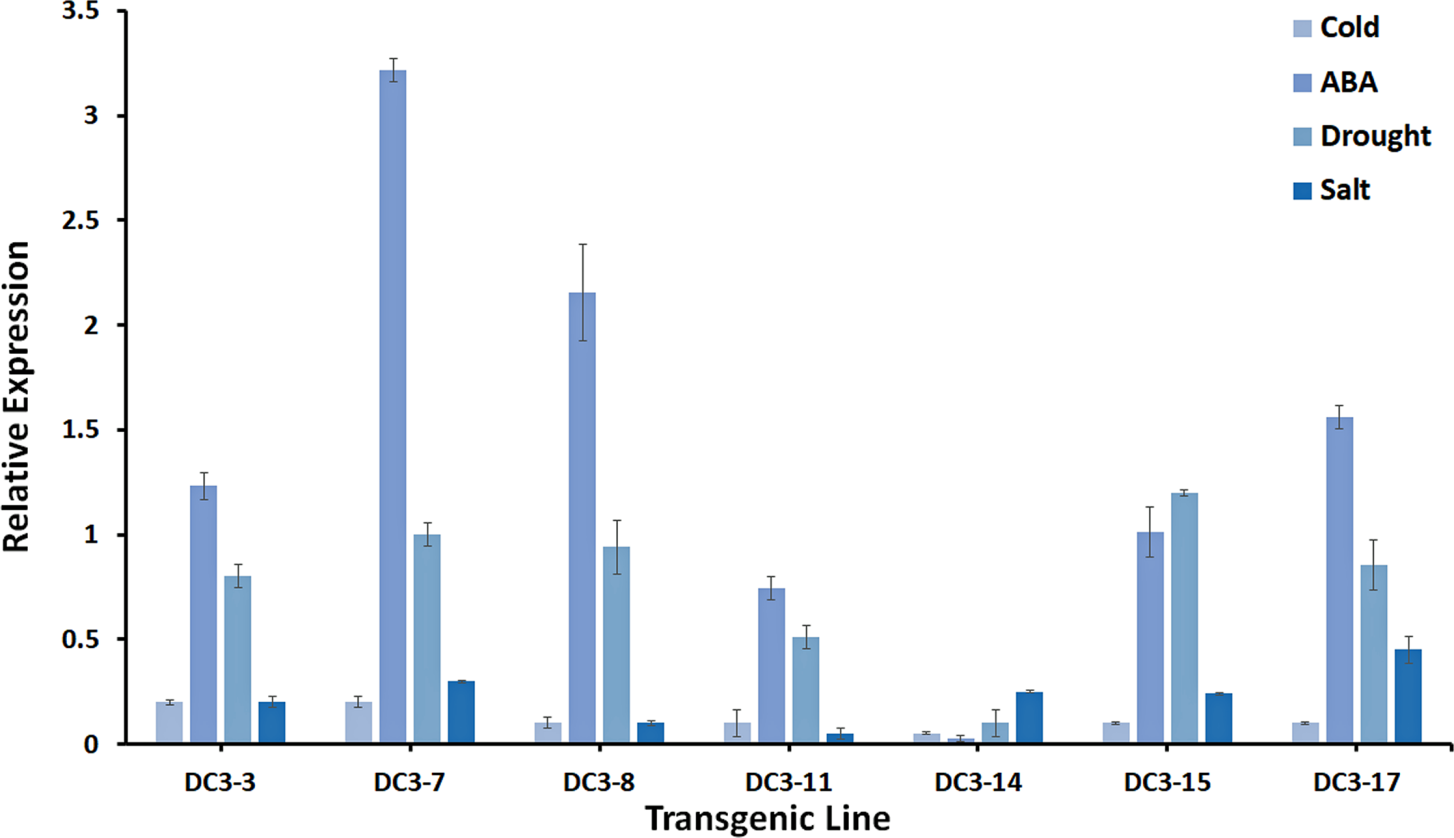
Relative *VvMybA1* gene expression in response to various abiotic stress imposed on the selected transgenic lines.

The *Dc3* promoter responds to several external stress stimuli [49]. The regenerated *Dc3* transgenic citrus plants were subjected to several stresses, and the relative gene expression of the *VvMybA1* transgene was evaluated in 6 independent transgenic lines. Cold stress treatment did not result in any significant effect on transgene expression in all lines evaluated (Fig 4). Relative gene expression following water stress treatments increased and ranged from a 1.2-fold change in DC3-15 to 0.1-fold change in DC3-14 (Fig 4). ABA application resulted in the greatest change in expression levels in all transgenic lines, except the line DC3-14, which did not respond to exogenous ABA application. A 3.2-fold change in gene expression level was observed in the line DC3-7, while the other lines had a 2.1- to 0.7-fold change in expression levels. Two drenches of 200 mM NaCl applied weekly induced *VvMybA1*expression, again in all lines except DC3-14. The *VvMybA1* expression levels ranged from 0.45-fold expression from line DC3-17 to 0.1-fold in DC3-8.

### Transgenic lines show similar levels of physiological responses compared to non-transgenic control

**Fig 5:**
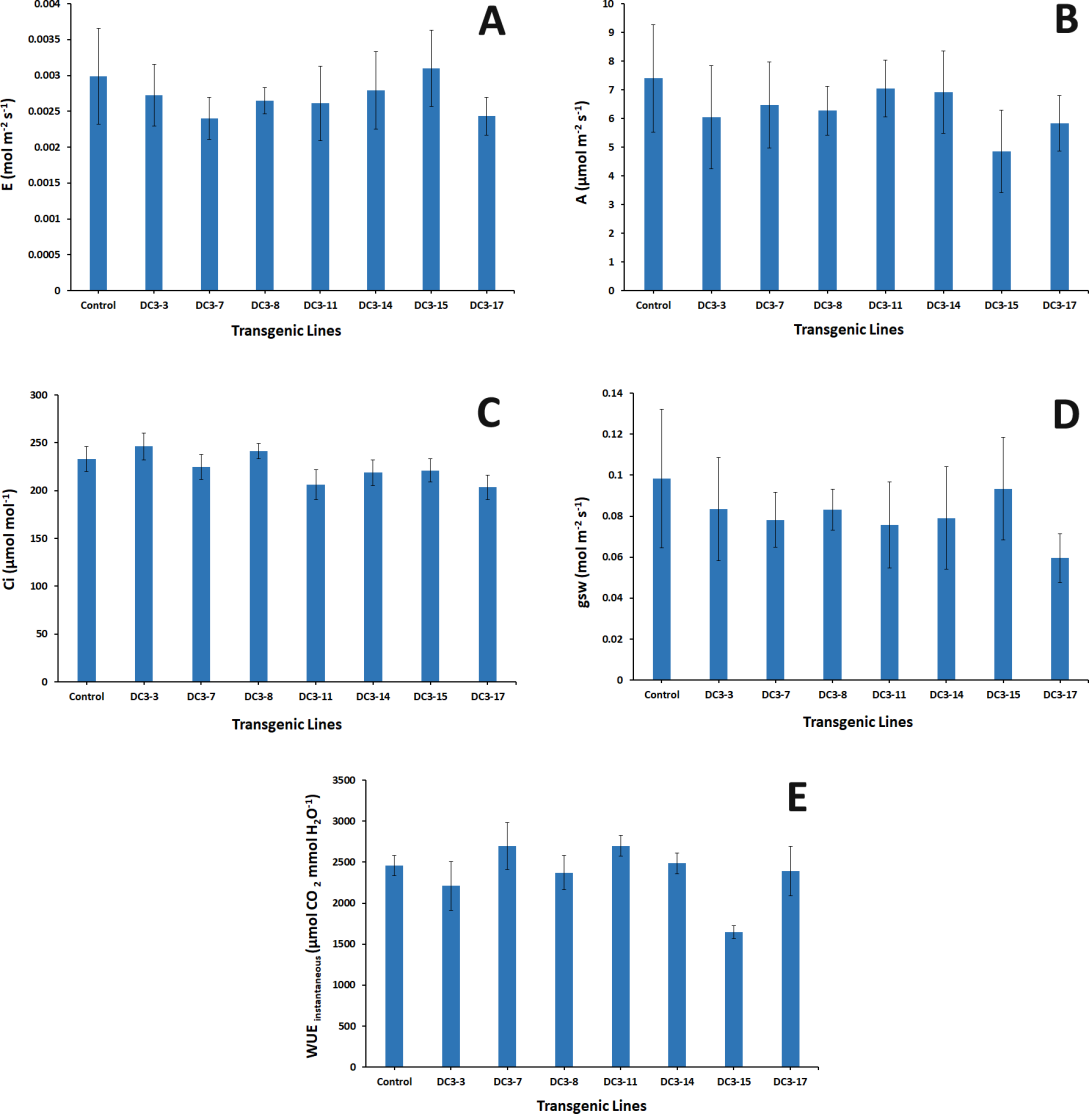
Comparison of several physiological parameters between selected transgenic lines and control. A) Transpiration rate; B) Net assimilation rate; C) Intercellular CO2 concentration; D) Stomatal conductance and E) Instantaneous physiological water use efficiency.

Several physiological parameters were evaluated in the regenerated transgenic lines. None of the transgenic lines differed from the non-transgenic control for net assimilation of CO_2,_ transpiration rate, intercellular CO_2_ content and stomatal conductance to water vapor responses. The WUE_instantaneous_ (a molar ratio of assimilation to transpiration) in transgenic line DC3-15 significantly differed from the control. All other transgenic lines were not significant (Fig 5)

## Discussion

The *Dc3* gene isolated from carrot (*Daucus carota* L.) is a group III lea-class gene [50] that is highly expressed during the initial phases of embryogenesis [51]. Its promoter is expressed in developing seeds [52] and can therefore be utilized to target seed specific transgenes or, as in our study, to drive a visual reporter gene to select transgenic cells following genetic transformation. Citrus can be efficiently transformed using cell suspension cultures and regenerated through somatic embryogenesis [53]. In addition, cell suspension cultures allow for the genetic transformation of any polyembryonic citrus cultivar that can be established as an embryogenic cell suspension culture, including the otherwise hard-to-transform mandarins and seedless cultivars that cannot be transformed through conventional epicotyl mediated transformation techniques [38].

The genetic transformation process relies heavily on the ability to select transformed cells from most non-transformed cells. This is because, in most plant species, DNA integration into the genome following transformation usually occurs at a low frequency [54]. Transgenic plants are commonly selected following the expression of a selectable marker gene linked to the gene of interest [55]. Thus, selection of transformed cells for their ability to proliferate in the presence of the selective agent allows isolation of transgenic cells containing only the gene of interest [56]. Once a desired transgenic plant is produced from transgenic cells, the marker gene becomes useless [57]. However, recent studies have focused on excision systems for the removal of the marker gene [58–60].

In our current study, we tested the *VvMybA1* anthocyanin regulatory gene (isolated from the grapevine) driven by the embryo-specific *Dc3* promoter as a regulated visual selectable marker for the selection of transgenic citrus lines. Anthocyanin regulatory genes have been used for the selection of transgenic cells in other plant species [61–63]. In most cases, these studies have relied on the utilization of a strong constitutive promoter to drive the anthocyanin regulatory gene. This invariably resulted in high anthocyanin accumulation in the cells and the regenerated plants exhibited growth retardation when compared to the non-transgenic plants [62]. The *Dc3* promoter is a weak promoter compared to the commonly used virus derived constitutive promoters and produces a much weaker gene expression pattern (our observations). Transgene expression under the control of the *Dc3* promoter did not produce high levels of anthocyanins in the embryo and we did not observe any developmental differences between transgenic and non-transgenic embryos. In apple, transgenic plants were obtained after visual selection with a construct containing the Myb10 gene driven by its own promoter. This resulted in much lower gene expression and the production of phenotypically normal plants [63].

This system also enabled the selection of transgenic embryos that could be easily identified by their purple coloration from the non-transformed green embryos (Fig 1C). The construct was initially tested using conventional *Agrobacterium*-mediated transformation–a process that requires an antibiotic selection step. Transgenic embryos identified visually based on color could be regenerated into normal plants without any visual coloration (Fig 1). Our results demonstrated the specific activation of the *Dc3* promoter in citrus embryo.

The protoplast expression vector was constructed to test an all-plant DNA sequence as a reporter. Protoplast transformation utilizing naked DNA offers the simplest technique to create an all-plant DNA-containing transgenic or even a cisgenic/intragenic plant. An added advantage of utilizing a visual marker with protoplast transformation is that it does not depend on a plant antibiotic-selectable marker for plant regeneration [64]. Although we utilized a plasmid DNA containing an *E*. *coli* antibiotic selection backbone in this study, our results indicated that protoplast transformation can efficiently incorporate the construct and allow the regeneration of a phenotypically normal plant similar to that obtained using *Agrobacterium*-mediated transformation (Fig 2). Protocols can subsequently be developed to incorporate a linear all-plant DNA construct or eventually an all-citrus DNA construct (cisgenic or intragenic) into the genome through protoplast-mediated transformation.

Chimeric transgenic plant production is a problem during citrus transformation and has been frequently observed with epicotyl-mediated transformation [65]. Chimeric plants arise due to multiple cells producing the shoot apical meristem–some cells are transformed and others are non-transgenic [66]. Somatic embryogenesis utilizes the ability of single cells to produce plants. A single-cell origin of somatic embryos has been reported in both monocots and dicots [67–70]. We did not regenerate any chimeric plants from the *Agrobacterium* mediated transformation system.

Protoplast transformation relies on the ability of PEG to agglutinate neighboring protoplasts and DNA [71]. During protoplast transformation, the cells are brought into close contact with the exogenous DNA, and a small amount of DNA is incorporated by endocytosis [72]. This process, however, also predisposes transient transformed cells in contact with neighboring untransformed cells to fuse and produce chimeric cell masses. This occurs when two adjacent cells (one transformed and the other wild type) fuse to produce a chimeric plant. In rare instances, two transformed cells (each containing a different copy number) can fuse to regenerate a visually non-chimeric plant. We observed the formation of a few chimeric embryos during protoplast transformation but they were not regenerated and subsequently discarded.

*VvMybA1* gene expression was low to negligible in the transgenic plants (Fig 3). Acid methanol extracts also did not produce a discernable change in coloration in these tissue samples (results not shown). Anthocyanins are a major component of many fruits [73] and vegetables [74]. There have been detailed studies on the safety of anthocyanins [75], and anthocyanins present in grape juice, wine and fruits (mostly produced as a result of the *VvMybA1* gene) have been consumed by humans for several millennia [76]. The anthocyanin selection system is comparable to the GUS and the GFP selection systems in grapes [77]. While GUS is used as a destructive marker [78], GFP is considered non-destructive [79]. Even though marker genes have not been implicated in any safety issues [80–82], it is our understanding that an anthocyanin-based marker would be safer and more consumer friendly than either GUS or GFP. Anthocyanin regulatory genes have been utilized as visual markers in other crop systems with variable success [83–85]. However, these studies relied on the use of a constitutive promoter that made practical application meaningless.

The *Dc3* promoter is influenced by external abiotic stimuli [49, 86], and we observed a variable response to different external stimuli. Exogenous ABA application produced the greatest change; this was expected since the promoter is ABA inducible. Since citrus trees are grafted, we did not test gene expression in the roots following treatment. The *Dc3* promoter is highly inducible in transgenic tobacco seedlings by salt and water stress [86]. The lower expression levels observed in our transgenic lines could be crop specific, with citrus being a woody perennial in comparison to published results from herbaceous seedlings [49, 52, 86]. It is possible to affirm that even though *VvMybA1* gene expression under the control of the Dc3 promoter was variable in response to different abiotic stresses, it was not highly induced in response to any stress. Thus, *VvMybA1* expression is virtually suppressed, and the induced levels are low to negligible under the normal stress that plants usually suffer. This is important to maintain a lack of marker gene expression in transgenic plants. GAPC was used as the reference gene since it has been thoroughly evaluated in citrus and deemed to a superior housekeeping gene for qPCR studies [43].

To understand if the *VvMybA1* gene had any physiological influence on the transgenic plants, we used an open gas exchange system, the LI-6800, which rapidly measures changes in the gas concentration using infrared gas analyzers (IRGAs) [87]. Measuring gas exchange in intact leaves is a precise and reliable method to evaluate plant response to environment, disease pressure and input rate limitations [88–90]. The transgenic lines behaved similarly when compared to controls under the same environment, and these results agree with citrus physiology characteristics and response to changes in environment [91–93]. The addition of the *VvMybA1* transgene did not change the transpiration rate, photosynthesis assimilation, CO_2_ assimilation rate or intercellular CO_2_ levels when compared to those of control non-transgenic trees (Fig 5). However, there was a significant change in the WUE_instantaneous_ in one of the transgenic lines which can be attributed to the lower photosynthesis rate reading in that line [94].

## Conclusion

Our research provides conclusive evidence supporting the utilization of a plant-derived, embryo-specific visual reporter system for the genetic transformation of citrus. It is possible that there could be transgene expression in the seeds, but due to the long juvenile phase in citrus, it was not possible to study gene expression in the fruit in the current study. However, there is an increasing demand for the cultivation of seedless citrus [95]. Most of the commercially available fresh-market citrus, such as clementines [96], Tango mandarin [97], and navel oranges [98], are already seedless. In addition, juice oranges are processed in specialized facilities where it would be possible to discard seeds before they reach the consumers. This coupled with the utilization of an anthocyanin regulatory gene as a marker may alleviate safety concerns among the producers, processors and consumers. In addition, identification of similar tightly regulated, embryo-specific promoters from the citrus genome coupled with citrus-derived anthocyanin regulatory genes would result in the creation of an intragenic component for the genetic transformation of citrus.

## Supporting information

S1 Fig DNA maps of the *Agrobacterium* and protoplast vectors used in this study. (PDF)

S2 Fig Cells used in the transformation experiment. A) Embryogenic citrus callus B) Citrus suspension cultures C) Protoplast ring in a sucrose-mannitol gradient following enzymatic digestion of suspension derived cells.
(TIF)

S3 Fig PCR results from transgenic lines regenerated from either cell suspension transformation (A) or protoplast transformation (B) and successfully acclimated to a greenhouse.
(TIF)
