# Supplementary figures and images for: Embryo-specific expression of a visual reporter gene as a selection system for citrus transformation
Source: PLoS One. 2018 Jan 2;13(1):e0190413. doi: 10.1371/journal.pone.0190413 (PMC5749800; doi:10.1371/journal.pone.0190413)

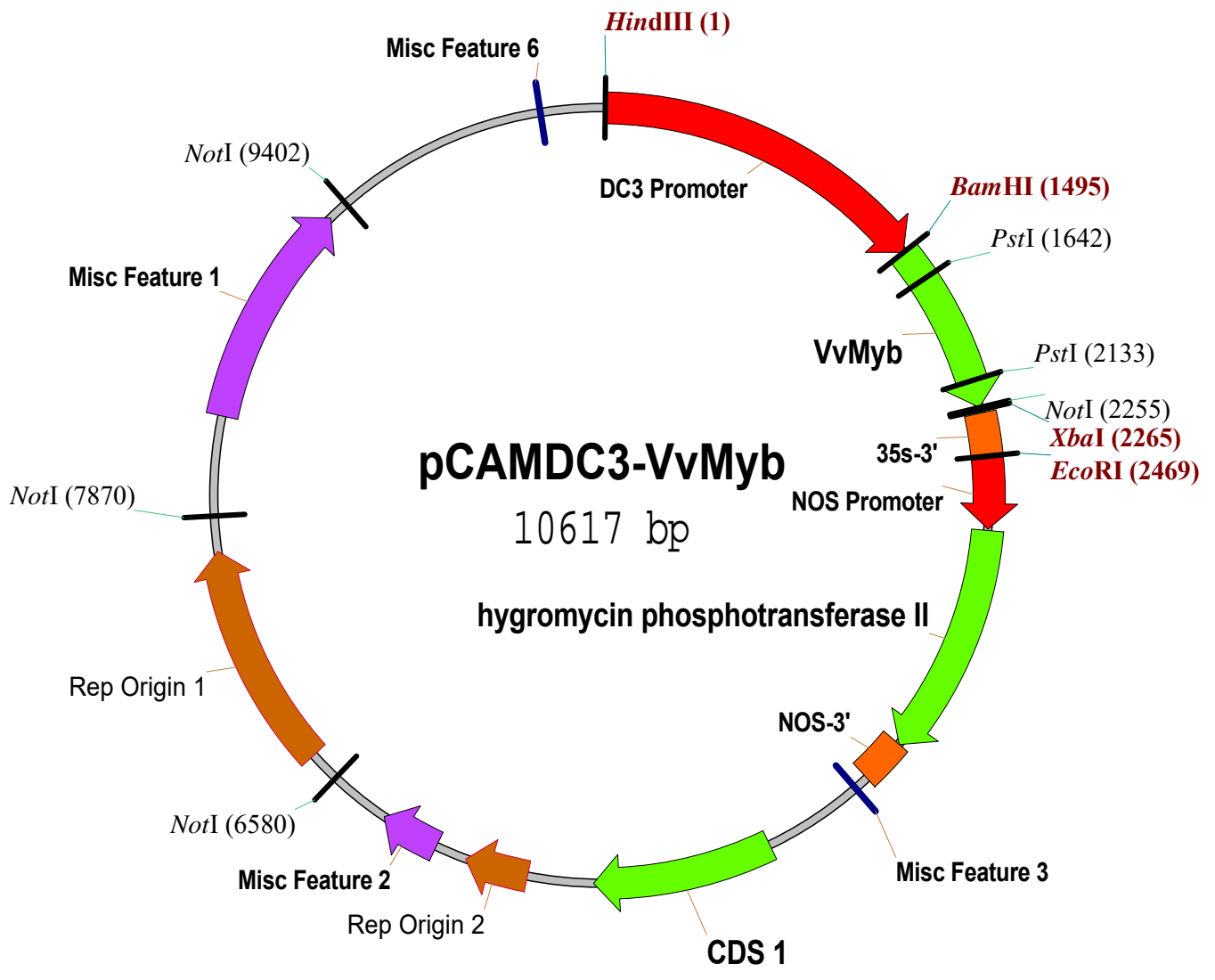

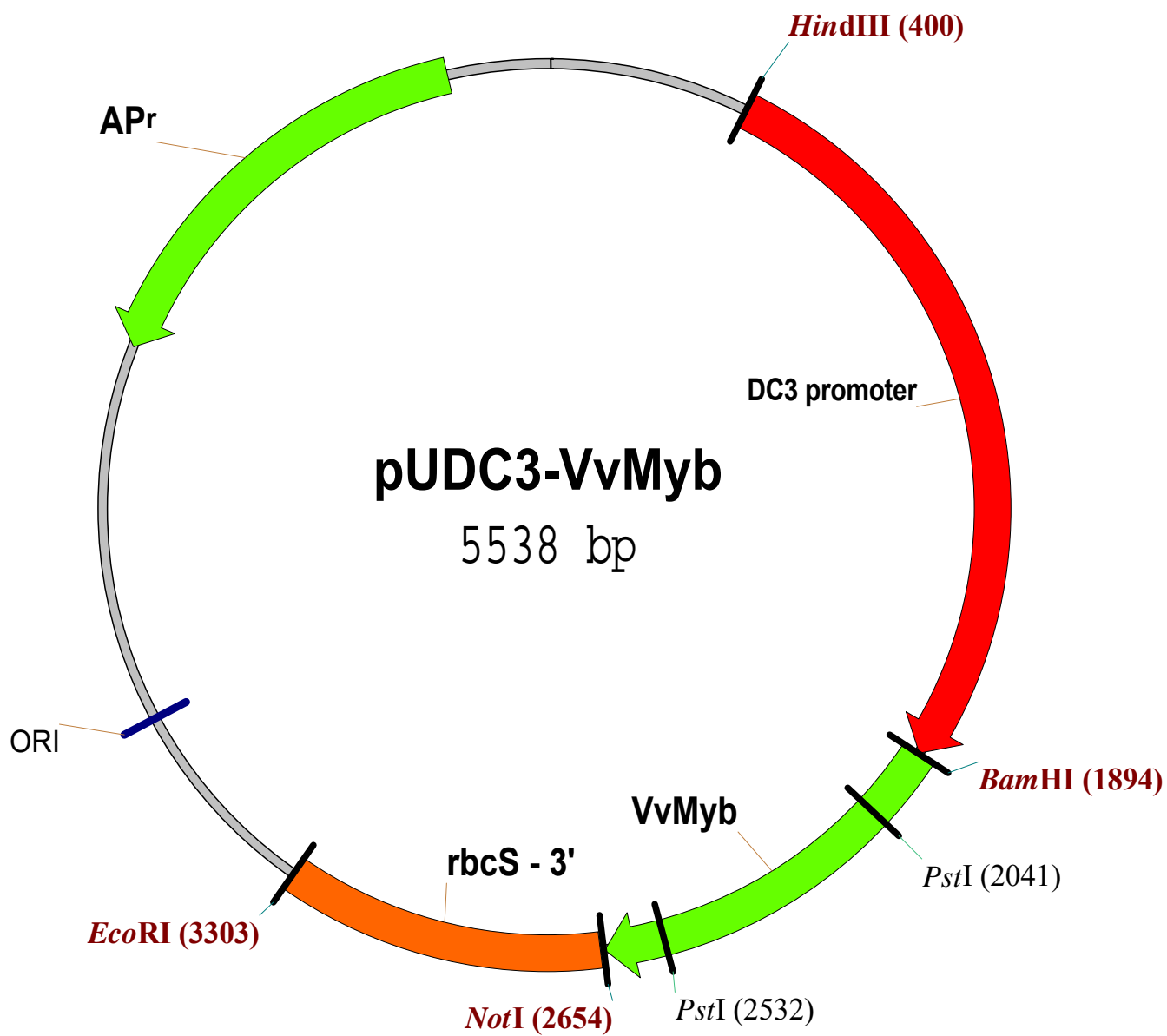

Supplement: S1 Fig — (PDF) [file pone.0190413.s001.pdf]

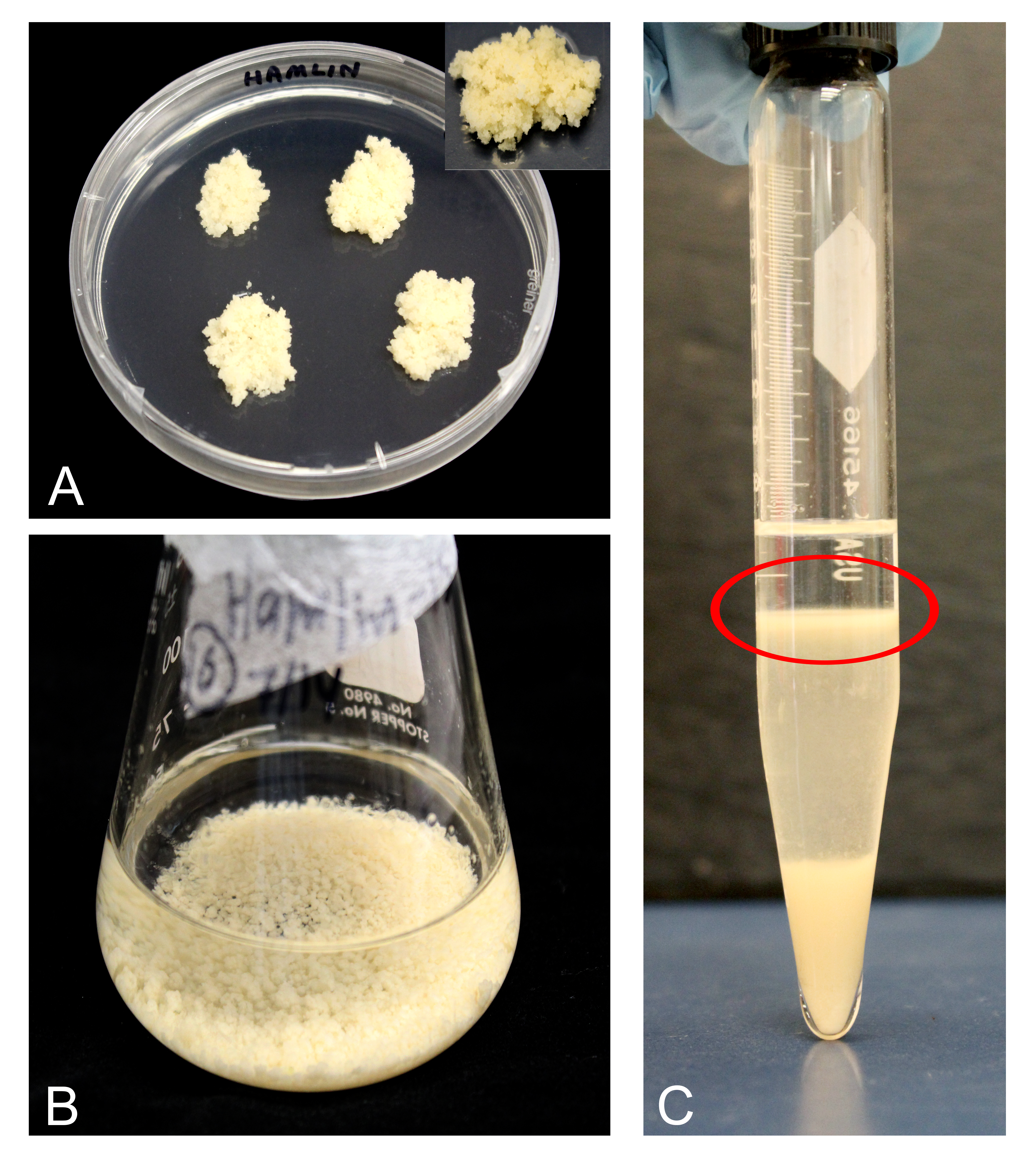

Supplement: S2 Fig — Cells used in the transformation experiment. A) Embryogenic citrus callus B) Citrus suspension cultures C) Protoplast ring in a sucrose-mannitol gradient following enzymatic digestion of suspension derived cells. (TIF) [file pone.0190413.s002.tif]

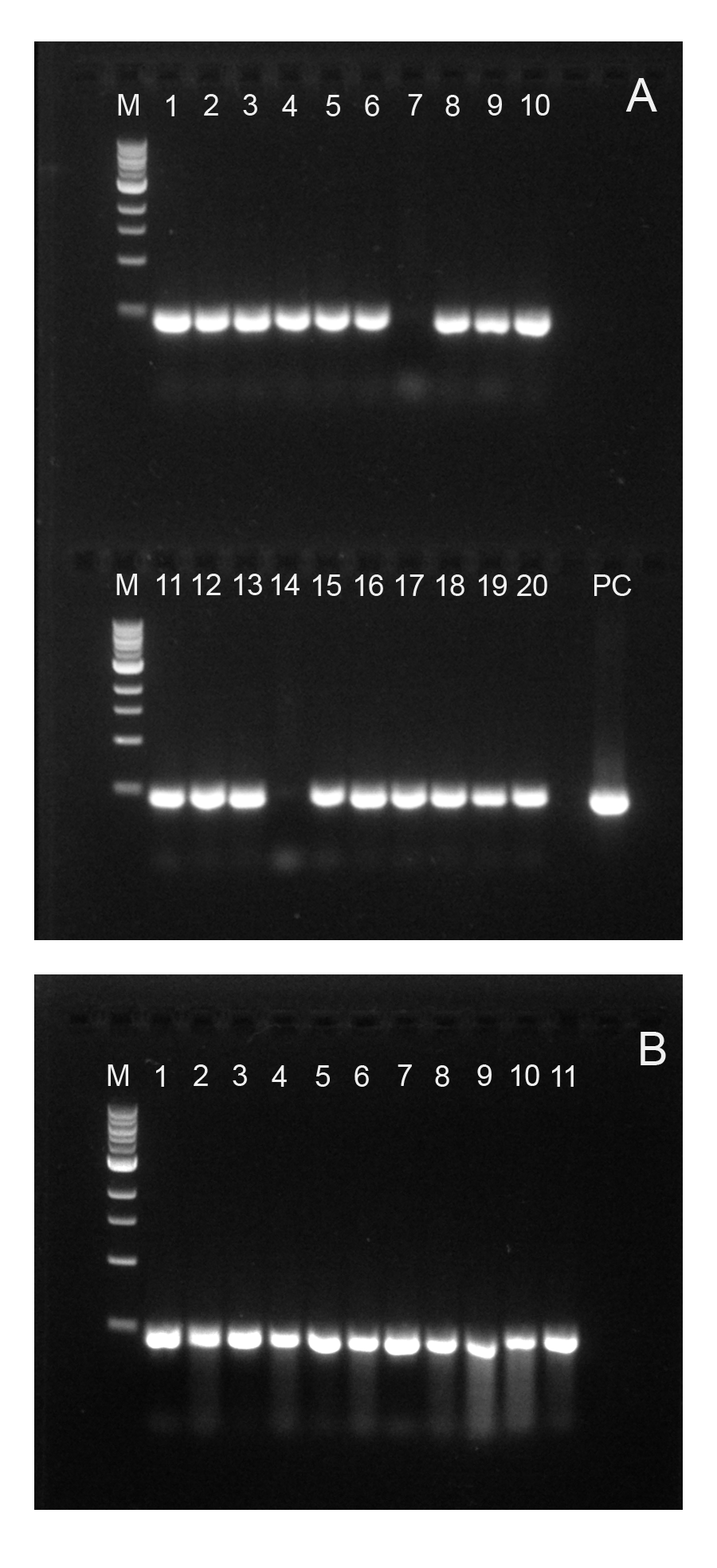

Supplement: S3 Fig — PCR results from transgenic lines regenerated from either cell suspension transformation (A) or protoplast transformation (B) and successfully acclimated to a greenhouse. (TIF) [file pone.0190413.s003.tif]
